# Canadian Prostate Cancer Trends in the Context of PSA Screening Guideline Changes

**DOI:** 10.3390/curroncol32120669

**Published:** 2025-11-29

**Authors:** Anna N. Wilkinson, Larry F. Ellison, Shary X. Zhang, Michael Ong, Scott C. Morgan, S. Larry Goldenberg, Rodney H. Breau, Christopher Morash

**Affiliations:** 1 Department of Family Medicine, Faculty of Medicine, University of Ottawa, Ottawa, ON K1H 8L6, Canada; 2 Centre for Population Health Data at Statistics Canada, Government of Canada, Ottawa, ON K1A 0T6, Canada; larry.ellison@statcan.gc.ca (L.F.E.); sharyxinyu.zhang@statcan.gc.ca (S.X.Z.); 3 Division of Medical Oncology, The Ottawa Hospital Cancer Centre, University of Ottawa, Ottawa, ON K1H 8L6, Canada; mong@toh.ca; 4 Division of Radiation Oncology, The Ottawa Hospital Cancer Centre, University of Ottawa, Ottawa, ON K1H 8L6, Canada; smorgan@toh.ca; 5 Department of Urologic Sciences and the Vancouver Prostate Centre, University of British Columbia, Vancouver, BC V5Z 1M9, Canada; l.gold@ubc.ca; 6 Division of Urology, Department of Surgery, University of Ottawa, Ottawa, ON K1H 8L6, Canada; rbreau@toh.ca (R.H.B.);

**Keywords:** prostate cancer, PSA, screening, mortality, cancer registry, stage, survival

## Abstract

Prostate cancer is the most diagnosed cancer among Canadian men, except non-melanoma skin cancers. Prostate-specific antigen (PSA) screening can enable early detection, but it is not formally recommended in Canada. Nonetheless, opportunistic screening persists, influenced by US practices and some Canadian guidelines that support screening. This study provides a detailed analysis of trends in prostate cancer incidence, mortality, stage distribution, and net survival in the context of evolving PSA screening guidelines. Prostate cancer case (1984–2022) and death (1984–2023) data were primarily from the Canadian Cancer Registry and the Canadian Vital Statistics Death database, respectively. Joinpoint regression identified incidence and mortality trends. Net survival was determined using the Pohar Perme estimator. Following the introduction of PSA screening, the prostate cancer age-standardized incidence rate among men aged 50–74 increased 1.8% annually until 2007 (*p* = 0.006) before declining at an annualized rate of −5.9% (*p* = 0.005) until 2014. Among men aged ≥75, incidence declined at −3.2% annually from 1992 to 2015 (*p* < 0.001). The prostate cancer age-standardized mortality rate (ASMR) among men aged 50–74 fell at an annualized rate of −4.3% between 1994 and 2010 (*p* = 0.022), but the decline slowed thereafter. The ASMR among men aged ≥75 continuously declined after a peak in 1995, with the greatest change noted before 2012. From 2010 to 2017, stage IV prostate cancer incidence increased across all 10-year age groups, peaking among men aged 60–69 at 4.1% annually (*p* < 0.001). Among men aged 50–74, the corresponding annualized increase was 3.7% (*p* = 0.010), while among men aged ≥75, it was 3.1% (*p* < 0.001). Although stage IV net survival among men aged 50–74 increased from 49.4% in 2010–2011 to 56.6% in 2016–2017, all-stage net survival declined slightly after 2011, concurrent with a shift towards a greater proportion of stage IV cases. Prostate cancer outcomes in Canada reflect US PSA screening recommendations. Organized and thoughtful screening may represent an opportunity to decrease the rising late-stage incidence.

## Simple Summary

Prostate cancer trends in Canada were associated with US PSA screening recommendations. After prostate cancer screening became widespread, cases increased rapidly in younger men and began decreasing among men aged 75 and older. Prostate cancer in Canada changed after the introduction of PSA screening, shifting the initial diagnosis to an earlier age. Prostate cancer mortality dropped by over 50% after screening started, with the biggest changes seen among men in their late 50s and 60s. After US recommendations against screening, mortality reductions lessened, and the number of early-stage cases decreased, while stage IV (metastatic/incurable) cancers increased among both younger and older men. Multiple new life-prolonging treatments for patients with metastatic disease have increased the survival for stage IV cancer. However, overall survival decreased from when screening was recommended, attributable to the increased number of incurable cases.

## 1. Introduction

Prostate cancer is the most common cancer in Canadian men aside from non-melanoma skin cancers. A projected 27,900 cases were expected to be diagnosed in Canada in 2024, accounting for 22% of all cancers among males [1]. Prostate cancer is the third leading cause of cancer deaths in Canadian men, ranking only behind lung and colorectal cancers, and is responsible for 11% of all cancer deaths in men.

**Figure 1:**
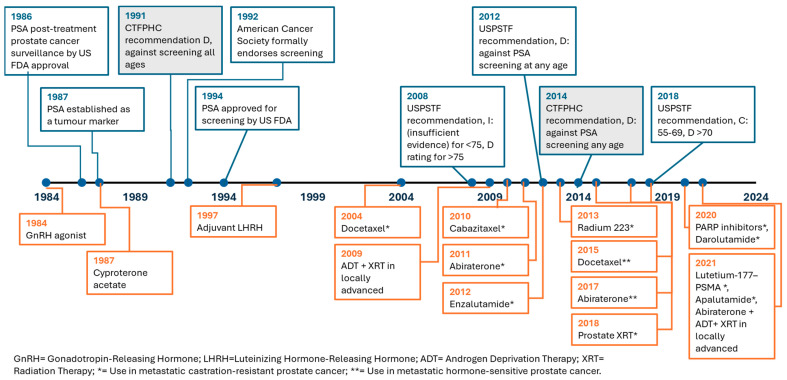
Timeline of prostate cancer screening and treatment innovations.

The treatments for prostate cancer have improved over time (Figure 1). Many patients with non-metastatic disease can now be safely observed without treatment (active surveillance). Less invasive surgical options have been developed, and refined radiation techniques have been adopted for those who need treatment [2,3,4,5,6]. Androgen deprivation therapy was introduced in 1984 [7]. In addition to surgical castration or GnRH-directed androgen deprivation, docetaxel chemotherapy was proven to be effective in 2004, and cabazitaxel emerged in 2010 as a second-line chemotherapy [8]. Additional agents targeting androgen synthesis (abiraterone acetate) and androgen receptor inhibition (enzalutamide, apalutamide, and darolutamide) were proven effective starting in 2011 [9,10]. The radioligand therapies Radium-223 and Lutetium-177 prostate-specific membrane antigen (PSMA) moved into clinical practice in 2013 and 2021, respectively [11,12]. Poly (ADP-ribose) polymerase or PARP inhibitors were also proven to be beneficial for some patients in 2020 [13].

Serum prostate-specific antigen (PSA) can be used to screen for prostate cancer, diagnosing cancers at an earlier stage when treatments are less intensive and more likely to be curative. The value of PSA screening is debated, as there is potential for overdiagnosis, especially in elderly men with multiple co-morbidities and indolent disease. Overtreatment can lead to significant side effects such as erectile dysfunction, bowel dysfunction, and urinary toxicity, which may deleteriously impact quality of life [14].

Several trials have investigated the impact of PSA screening on prostate cancer mortality. The Prostate Lung Colorectal Ovarian (PLCO) trial showed no significant reduction in prostate cancer mortality with screening. However, this trial had a significant limitation, with nearly 90% of men in the control arm undergoing PSA testing. As a result, the PLCO trial was limited in its ability to determine the benefit of screening [15,16]. The European Randomized Study of Screening for Prostate Cancer and the Göteborg trials demonstrated relative mortality reductions of 21% and 44% at 13 and 14 years post diagnosis, respectively, while screening in the Kaiser Permanente retrospective cohort study was associated with a 64% reduction in prostate cancer mortality over 16 years [17,18,19]. The number of men needed to screen to prevent a death from prostate cancer improved with a longer follow-up period, from 1410 after 9 years to 570 after 16 years, and to 456 after 23 years [20]. An absolute reduction of 3.1 cases of metastatic disease per 1000 men is noted with screening [21]. Notably, participation in major prostate cancer trials has been 96% among white men, so the observed results may not be generalizable to other races and ethnicities [22,23,24].

A 1987 study of PSA in patients with prostate cancer established its use as a tumor marker (Figure 1) [25]. The seminal paper describing the utility of PSA for screening was published in 1991, and it was approved for use in screening by the United States (US) Food and Drug Administration in 1994 [26]. The Canadian Task Force on Preventive Health Care (CTFPHC) recommended against PSA screening in 1991 and again in 2014, while Canadian specialty societies have continued to recommend screening for those in good health with a long life expectancy [27,28]. Screening was widely used in the United States until 2008, when the US Preventive Services Task Force (USPSTF) suggested that there was insufficient evidence for PSA screening in men younger than 75 and recommended against screening in men older than 75. In their draft recommendation in 2011, finalized in 2012, the USPSTF recommended against PSA screening in men of all ages [29]. However, in 2018, they issued a neutral recommendation for men aged 55–69 and continued to recommend against screening for men older than 70 [30]. Access to PSA screening in Canada remains variable, as it is publicly funded in some provinces if ordered by a primary care provider, while it is self-pay in Ontario, British Columbia and Quebec. Maintaining the benefits of PSA screening while mitigating potential harms has been an ongoing area of study in Canada. The use of Magnetic Resonance Imaging (MRI) as a secondary screening test reduces both the number of biopsies needed and improves the detection of higher-risk cancers [31,32,33].

Despite consistent CTFPHC recommendations against prostate cancer screening, some Canadian men have continued to access screening. A study using the Canadian Community Health Survey found that the prevalence of lifetime PSA screening rates in Atlantic Canada, Ontario, and Quebec increased from 2001 to 2013, with over half of men having at least one PSA in their life, and ongoing screening activity was noted in men older than 70 [34]. There is evidence of decreasing PSA uptake in more recent years, with a 56% relative reduction in PSA screening noted in one Ontario health region from 2011 to 2015 [35]. In the US, a patient survey documented an 18% relative reduction in PSA screening rates between 2010 and 2013 [36], and a 23.4% relative decline in a regional health care system between 2010 and 2015 [37].

After the USPSTF recommendations against screening in the US, prostate cancer incidence in the US decreased, while advanced stage at diagnosis and prostate cancer mortality increased [38]. Although screening has never been recommended in Canada by the CTFPHC, the proximity of Canada to the US has knock-on impacts on screening activities in Canada [39]. This study provides a comprehensive examination of Canadian prostate cancer incidence, mortality, stage at diagnosis, and net survival in the context of PSA screening guideline changes.

## 2. Methods

This ecological study included prostate cancer case counts for 1992 to 2022 from the Canadian Cancer Registry (CCR) file released 31 January 2025 [40], and case counts from 1984 to 1991 from the CCR’s predecessor, the National Cancer Incidence Reporting System (NCIRS). For the CCR data, prostate cancer cases were defined as site code C619 (excluding histology types 9050 to 9055, 9140, 9590 to 9993) based on the First Revision of the International Classification of Diseases for Oncology, Third Edition [41]. For the NCIRS data, they were defined as code 185 according to the International Statistical Classification of Diseases and Related Health Problems, Ninth Revision (ICD-9) [42]. Prostate cancer death counts for 1984 to 2023 were obtained from the Canadian Vital Statistics—Death Database (CVSD) file released 19 February 2025 [43]. Prostate cancer deaths were defined according to ICD-9 [42] prior to 2000 and ICD-10 [44] thereafter. Population data were from Statistics Canada [45]. This study was a secondary analysis of nationally deidentified data collected by Statistics Canada, and, as such, ethics approval was not required.

Incidence data from Quebec were excluded for multiple reasons. For data years prior to 2011, submissions to the CCR from Quebec relied primarily on hospital data (i.e., hospitalizations or day surgeries) to identify diagnosed cancers. As such, the number of cases of some cancers, particularly prostate and skin melanoma, is considerably underestimated for these years [46]. Cases diagnosed in Quebec from 2018 onward are unavailable as they had not been submitted to the CCR. Finally, no staging information was available for Quebec cases. Incidence analyses additionally exclude cases diagnosed in Nova Scotia for 2020 onward, as these cases had also not been submitted to the CCR. As no death certificate only cases had been reported to the CCR from Manitoba from 2013 to 2017 and for 2022, from Ontario for 2022, from Newfoundland and Labrador for 2022, and from Nova Scotia for 2019, 95 such cases in total (Manitoba: 31, Ontario: 54, Newfoundland and Labrador: 1, Nova Scotia: 9) were imputed using methodology employed and described elsewhere [47,48].

Trends in age-specific and age-standardized prostate cancer incidence and mortality rates, and annual percent changes (APCs) for each identified trend were determined using the Joinpoint Regression Program [49]. A piecewise linear regression model that assumes a constant rate of change in the logarithm of the annual incidence rate was fitted [50].

An uncorrelated error model was selected for the autocorrelated errors option, and the Weighted Bayesian Information Criterion was used for model selection. *p*-values correspond to two-sided tests of the null hypothesis that the APC is zero, with a significance level of 0.05. No additional adjustments were made regarding multiple hypothesis testing. To match analytic techniques employed in recent Canadian Cancer Statistics publications, 1984 was chosen as the initial data year of analysis, the minimum time span on which to report a trend was set at five years, the maximum number of permitted joinpoints was five, and 2020 was removed as an anomalous data year for the incidence analysis arising from the COVID-19 pandemic [47,48]. Rates were age-standardized to the 2021 Canadian Standard Population [48,51].

Information to determine prostate cancer stage was only consistently available on the CCR since diagnosis year 2010 for all jurisdictions except Québec (all years), Saskatchewan (for 2018 only), Nova Scotia (for 2019 onwards), Newfoundland and Labrador (for 2021 onwards), and Ontario (for 2022 only). Consequently, these cancer cases and all cases diagnosed before 2010 or after 2021 were excluded from stage analyses. Stage at diagnosis for cancer cases diagnosed from 2010 to 2017 was determined according to the Collaborative Stage (CS) Data Collection System Version 2, which is based on the American Joint Committee on Cancer (AJCC) Cancer Staging Manual, 7th edition [52,53]. For cancer cases diagnosed from 2018 to 2021, stage at diagnosis was derived according to the AJCC Cancer Staging Manual, 8th edition [54]. As the AJCC 8 system distinguishes between clinical and pathological stage groups, the pathological stage group was used, if available. If the pathological stage group was unknown, the clinical stage group was used, if available. Among various changes from AJCC 7 to AJCC 8, AJCC 8 uses additional criteria to determine stage, namely, AJCC 8 adds tumor grade group. Under AJCC 8, stage IIIB and IIIC can now include cases that would have been staged as IV under AJCC 7.

Annual incidence rates for stage IV were presented separately for the 2010–2017 and 2018–2021 periods due to changes in staging systems, differences in completeness across jurisdictions and years, and sizeable differences in the proportion of cases that could not be assigned stages I to IV across systems. For the 2010–2017 period, 122,530 of 135,250 included cases (90.6%) among men aged 50 and older had sufficient information to be categorized as stages I to IV; the corresponding figures for 2018–2021 were 54,480 of 69,905 (77.9%). Joinpoint trends were computed only for the 2010–2017 period because fewer than five data points were available for analysis between 2018 and 2021. Stages I-III were combined because there is little variation in their respective five-year net survival estimates [55], and to enhance statistical power.

Net survival analyses were undertaken using a pre-existing analytic file created by linking cases from the CCR file (1992–2021) released 31 January 2024 [40], to mortality information, complete through 31 December 2021, via Statistics Canada’s Social Data Linkage Environment [56]. The mortality information was obtained from the CCR, the CVSD and the T1 personal master file (as reported on tax returns). For reasons detailed earlier for incidence data, cases diagnosed in Quebec and cases diagnosed in Nova Scotia in 2020 and 2021 were excluded from survival analyses. Provincial- and territorial-specific expected survival probabilities, necessary for the calculation of net survival in a relative survival framework, were obtained from Statistics Canada [57,58]. Where available, single-year population life tables were used. Expected survival probabilities used in the calculation of net survival for prostate cancer were adjusted for prostate cancer mortality rates in the general population [59,60].

Net survival estimates cancer survival in the hypothetical absence of other causes of death unrelated to the cancer diagnosis. Prostate cancer net survival estimates were derived using an algorithm [61] that has been augmented to include the Pohar Perme estimator of net survival [62] using the hazard transformation approach [63]. Cases with an undefined survival time (e.g., cases for which the diagnosis had been established through autopsy or death certificate only) were necessarily excluded. The period method was used to determine predicted net survival estimates for recent periods (e.g., 2019-to-2021) [64]. Two-sided statistical tests of the null hypothesis that net survival estimates were zero, with a significance level of 0.05, were performed on an ad hoc basis to support the description and interpretation of results.

## 3. Results

A total of 543,545 prostate cancer cases and 148,475 prostate cancer deaths among men aged 50 and older were considered in the incidence and mortality trends, respectively.

### 3.1. Age-Standardized Trends

Different patterns in the prostate cancer age-standardized incidence rate (ASIR) were observed between younger (50–74) and older (>75) men. While rates increased for both groups prior to the widespread availability of PSA screening in the early 1990s, they did so at a much-accelerated pace among the younger group (APC = 9.0%, *p* = 0.001 versus APC = 3.2%, *p* < 0.001) (Table 1, Supplemental Figure S1). After screening was available, the ASIR in the 50–74 group continued to rise, although more gradually (APC = 1.8%, *p* = 0.006), peaking in 2007. Beyond 2007, the ASIR decreased at an annualized rate of −5.9% (*p* = 0.005) until 2014. In contrast, the ASIR decreased at a rate of −3.2% per year (*p* < 0.001) from 1992 to 2015 among men aged 75 and older. No statistically significant trends in ASIRs were observed in either group in the most recent period. Overall, the ASIR dropped by 12.9% from 2019 to 2020 during the first calendar year of the COVID-19 pandemic.

**Figure 2:**
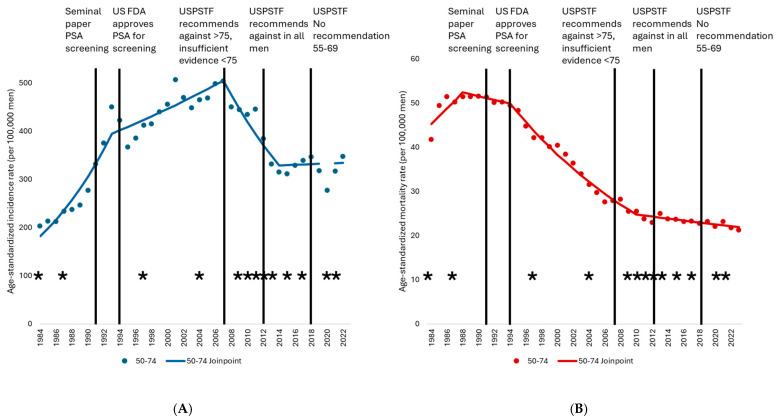
(**A**) Prostate cancer age-standardized incidence rates and trend models, males, 50–74, Canada excluding Quebec, 1984 to 2022. (**B**) Prostate cancer age-standardized mortality rates and trend models, males, 50–74, Canada, 1984 to 2023. Notes: Quebec was excluded from incidence because prostate cancer case ascertainment in this province significantly differed from other jurisdictions for much of the study period, and because cases diagnosed in this province from 2018 onward had not been submitted to the Canadian Cancer Registry (CCR). Incidence analyses additionally exclude cases diagnosed in Nova Scotia for 2020 onward, as these cases had also not been submitted to the CCR. Black bars represent key points in screening evolution. Black asterisks mark major systemic therapy changes with survival benefit. 1984: GnRH agonist; 1987: cyproterone acetate; 1997: adjuvant LHRH; 2004: Docetaxel in mCRPC; 2009: ADT + XRT in locally advanced; 2010: Cabazitaxel mCRPC; 2011: Abiraterone; 2012: Enzalutamide; 2013: Radium 223; 2015: Docetaxel mHSPC; 2017: Abiraterone mHSPC; 2018: XRT in low volume mHSPC; 2020: Olaparib, Darolutamide; 2021: Apalutamide, Lutetium-177–PSMA, Abiraterone + ADT + XRT in locally advanced. Sources: Canadian Cancer Registry (1992–2022), National Cancer Incidence Reporting System (1984–1991) at Statistics Canada, and Canadian Vital Statistics Death database at Statistics Canada.

Among men aged 50–74, the prostate cancer age-standardized mortality rate (ASMR) increased by nearly 4% per year (*p* = 0.001) from 1984 to 1988, declined slightly but non-statistically significantly (*p* = 0.077) over the next several years, and then decreased from 1994 to 2010 at an annualized rate of −4.3% (*p* = 0.022) (Table 1, Supplemental Figure S1). A more gradual decrease of −1.0% per year (*p* = 0.023) up to 2023 has subsequently been observed. A 59% decline in the prostate cancer ASMR among men aged 50–74 was achieved from 1990, when the rate peaked at 51.5 per 100,000 men, to 2023, when a low of 21.3 per 100,000 men was recorded. A 50% decline was noted between 1990 and 2010 alone. Men aged 75 and older experienced an increasing ASMR (APC = 1.4, *p* < 0.001) up to 1995, when the rate peaked at 512.7 per 100,000 men. The ASMR subsequently declined by 46% from 1995 to 2023, first by −2.5% (*p* = 0.001) annually until 2012—a 37% drop over this period—and then by −1.6% annually until 2023 (*p* = 0.006, 15% decrease). Age-standardized trends relative to screening and treatment changes are shown in Figure 2A,B.

### 3.2. Age-Specific Trends

**Figure 3:**
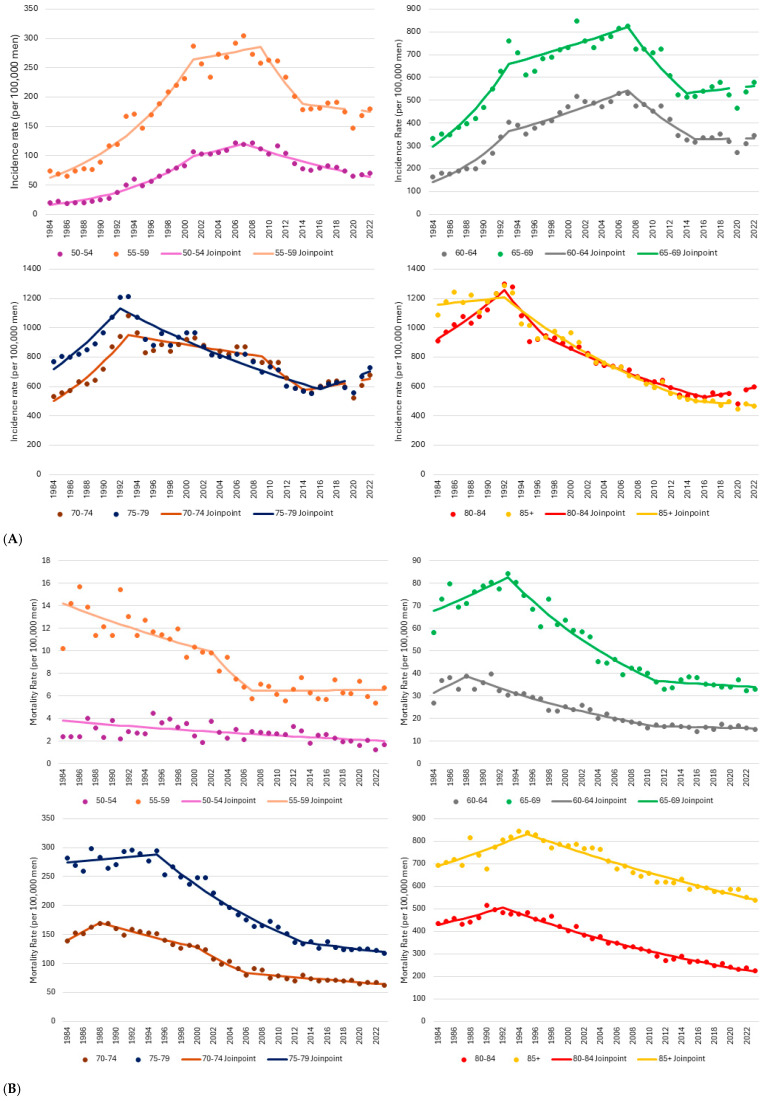
(**A**) Prostate cancer age-specific incidence rates and trend models, males, Canada excluding Quebec, 1984–2022. (**B**) Prostate cancer age-specific mortality rates and trend models, males, Canada, 1984–2023. Notes: The 2020 incidence data point is not connected to adjoining data points because the incidence data from this year were considered anomalous, arising from the COVID-19 pandemic. Quebec was excluded from incidence because prostate cancer case ascertainment in this province significantly differed from other jurisdictions for much of the study period, and because cases diagnosed in this province from 2018 onward had not been submitted to the Canadian Cancer Registry (CCR). Incidence analyses additionally exclude cases diagnosed in Nova Scotia for 2020 onward, as these cases had also not been submitted to the CCR. Sources: Canadian Cancer Registry (1992–2022), National Cancer Incidence Reporting System (1984–1991) at Statistics Canada, and Canadian Vital Statistics Death database at Statistics Canada.

From 1984 until the early 1990s (i.e., prior to the widespread availability of PSA screening), highly statistically significant increasing prostate cancer incidence rates were observed for each five-year age group between the age groups of 60–64 and 80–84. The pace of increase ranged from 11.0% per year (60–64) to 3.9% per year (80–84), with the APC monotonically decreasing with advancing age. Among men aged 50–59 strongly increasing trends of about 10% per year continued until 2001 (Table 2, Figure 3A). The age-specific prostate cancer peak incidence rate among men older than 70 occurred in the early 1990s, prior to the widespread availability of screening. Conversely, it peaked around 2007 among men aged 50–69, except for an aberrantly high rate among men aged 65–69 in 2001. Between the late 2000s and the mid-2010s, age-specific prostate cancer incidence rates among men aged 55–74 rapidly decreased by 6–8% per year (all *p* < 0.01). Among men aged 50–54, the rate of decrease was lower (APC = −4.2, *p* = 0.001), but continued through to 2022. For older age groups, incidence rates decreased by 3–4% per year from 1992 to the mid-2010s (all *p* < 0.005). Among men aged 80–84 specifically, a 59% decline in the incidence rate was noted between 1992 and 2016. However, between 2016 and 2022, incidence among men in this age group began to rise again (APC = 2.0%, *p* = 0.041).

Declines in prostate cancer mortality rates among men aged 50 to 54 spanned the study period (APC = −1.6%, *p* = 0.001), while significant declines among those aged 55 to 59 were restricted to the period from 2002 to 2007 (APC = −8.3%, *p* = 0.028) (Table 2, Figure 3B). In older age groups, excepting those aged 75 to 79, mortality rates initially increased until the late 1980s or early 1990s, highlighted by a 4.9% annualized increase from 1984 to 1988 among men aged 70 to 74 (*p* = 0.010). Over this same five-year period, a 5.4% per year increase among men aged 60 to 64 approached statistical significance (*p* = 0.051). Mortality rates then declined by about 4% per year among men in the 60 to 64, 65 to 69, and 75 to 79 age groups until 2011 to 2013 (all *p* < 0.001). Mortality declines after this time in these age groups flattened out and were non-significant. Reductions of about 60% were achieved in each of these age groups from the initiation of the respective downwards trends to 2023, with over 90% of the mortality reduction occurring during the period of significant change. Among men aged 70–74, the prostate cancer mortality rate has been significantly decreasing since 1988, with the largest decrease occurring between 2009 and 2014 (APC = −6.7, *p* = 0.009). Over the 35-year period, a 63% reduction in the mortality rate was achieved in this age group. Among men aged 80 and older, mortality rates have continued to decline by 2–3% per year for close to three decades (both *p* < 0.001). As a result, these rates have been reduced by 54% since 1992 among men aged 80–84, and by 36% since 1995 among men aged 85 and older.

### 3.3. Age-Specific Rate Patterns over Time

**Figure 4:**
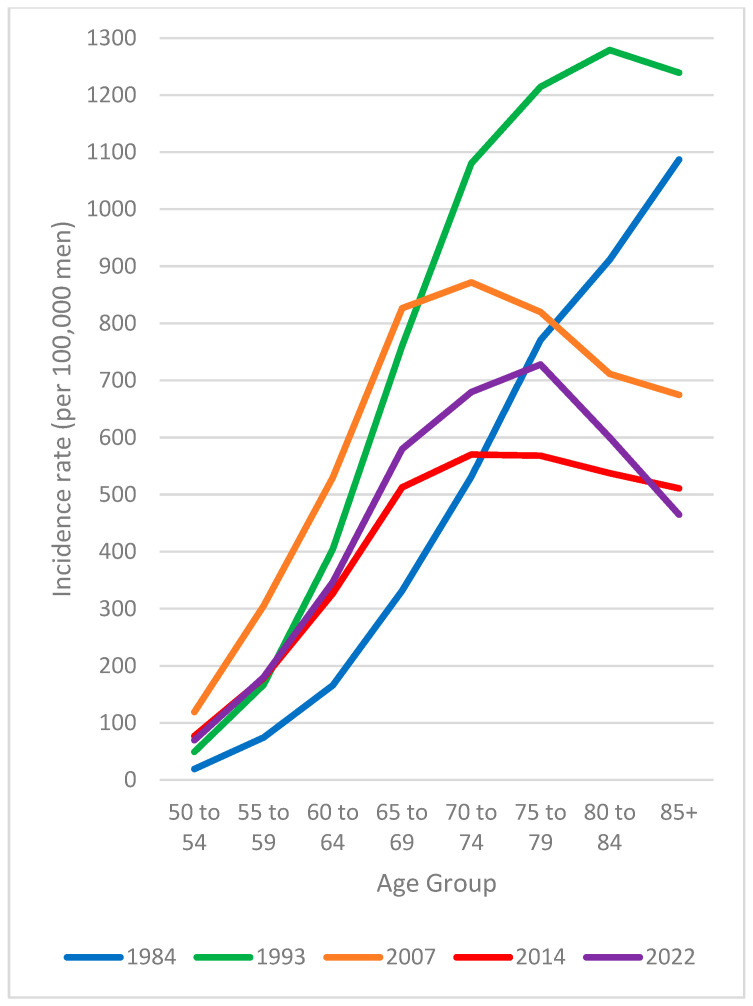
Prostate cancer age-specific incidence rates, males, Canada excluding Quebec, selected years. Notes: Quebec was excluded from incidence because prostate cancer case ascertainment in this province significantly differed from other jurisdictions for much of the study period, and because cases diagnosed in this province from 2018 onward had not been submitted to the Canadian Cancer Registry (CCR). Incidence analyses additionally exclude cases diagnosed in Nova Scotia for 2020 onward, as these cases had also not been submitted to the CCR. Sources: Canadian Cancer Registry (1992–2022), National Cancer Incidence Reporting System (1984–1991) at Statistics Canada, and Canadian Vital Statistics Death database at Statistics Canada.

With the evolution of age-specific prostate cancer incidence rates over time described above, the shape of the age-specific incidence rate curve has changed considerably over the years (Figure 4). In 1984, prostate cancer incidence rates per 100,000 men monotonically increased from a rate of 19.3 among men aged 50–54 to 1087.1 among men aged 85 and older. By 1993, rates were systematically higher and peaked in the 80–84 age group (1279.1). A decade and a half later, in 2007, rates were higher than seen in 1993 in men before the age of 70, but substantially lower in comparison afterwards, plateauing across the 65–69, 70–74, and 75–79 age groups with a peak among men aged 70–74 (872.0). By 2014, age-specific incidence rates among men aged 65–69 and older were considerably more consistent than at any other considered point and did not exceed 575 per 100,000 men in any age group. The most recent data from 2022 indicates that rates are now higher than they were in 2014 in men between the ages of 60 to 84, peaking among men aged 75–79 (728.0) and then sharply declining with age thereafter.

### 3.4. Stage Trends

**Figure 5:**
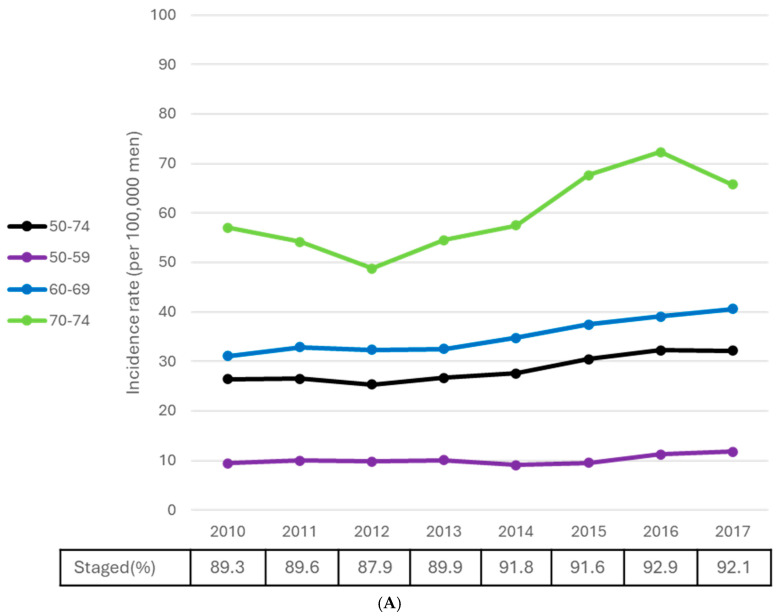

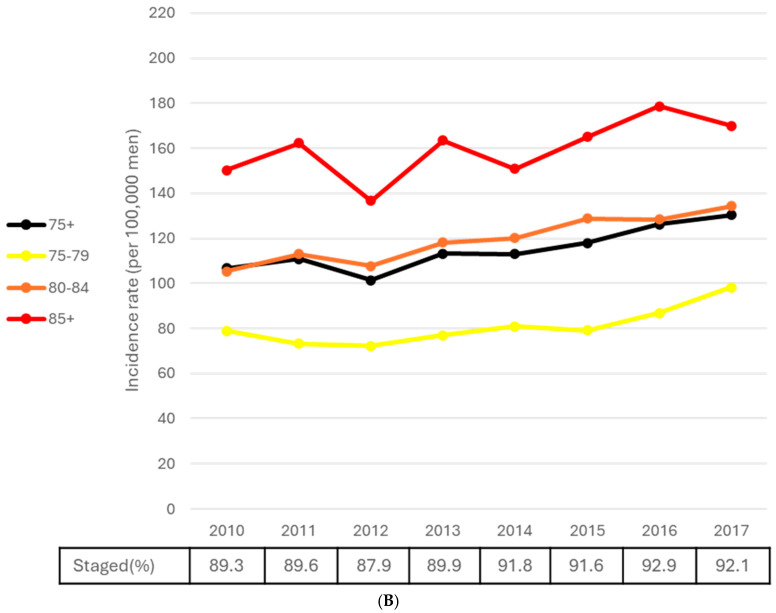
(**A**) Stage IV prostate cancer age-standardized and age-specific rates, males, ages 50–74, Canada excluding Quebec, 2010 to 2017. (**B**) Stage IV prostate cancer age-standardized and age-specific rates, males, ages 75 and older, Canada excluding Quebec, 2010 to 2017. Notes: The percentage staged refers to the percentage of total cases assigned stage I–IV exclusively. Quebec was excluded because prostate cancer cases submitted to the Canadian Cancer Registry, for this province and for this period, lacked staging information. Age-standardized rates (50–74 and 75+) were calculated using the 2021 Canadian Standard Population. Prostate cancer cases were staged using the Collaborative Stage (CS) data collection system, and American Joint Committee on Cancer (AJCC) 7th Edition stage groups were derived using CS data/variables. Source: Canadian Cancer Registry (1992–2022) at Statistics Canada.

**Figure 6:**
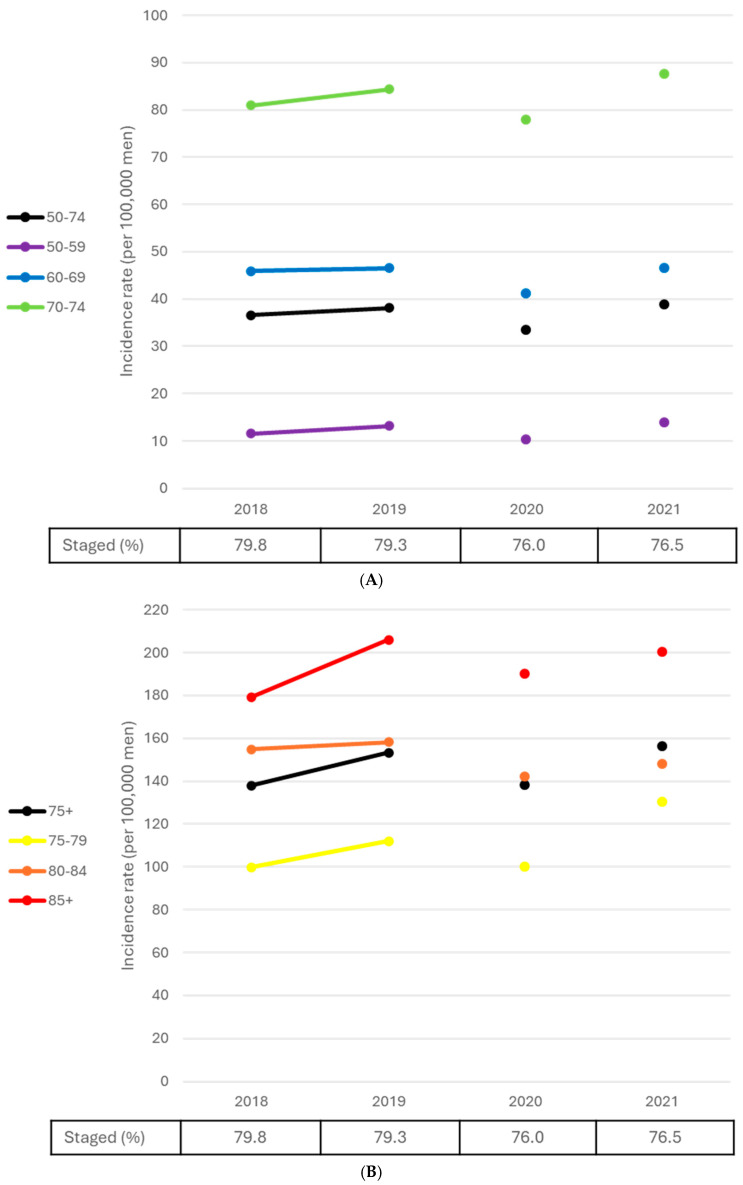
(**A**) Stage IV prostate cancer age-standardized and age-specific rates, males, ages 50–74, Canada excluding Quebec, 2018 to 2021. (**B**) Stage IV prostate cancer age-standardized and age-specific rates, males, ages 75 and older, Canada excluding Quebec, 2018 to 2021. Notes: The 2020 incidence data point is not connected to adjoining data points because the incidence data from this year were considered anomalous, arising from the COVID-19 pandemic. The percentage staged refers to the percentage of total cases assigned stage I–IV exclusively. Quebec was excluded because cases diagnosed in this province from 2018 onward had not been submitted to the Canadian Cancer Registry (CCR). Cases diagnosed in Nova Scotia for 2020 onward were similarly unavailable. Cases diagnosed in Saskatchewan in 2018, Nova Scotia in 2019, and Newfoundland and Labrador in 2021 were excluded because they lacked staging information. Age-standardized rates (50–74 and 75+) were calculated using the 2021 Canadian Standard Population. Prostate cancer cases were staged using the American Joint Committee on Cancer 8th Edition. Source: Canadian Cancer Registry (1992–2022) at Statistics Canada.

Statistically significant decreases in prostate cancer incidence rates for stages I–III combined from 2010 to 2017 were observed among men aged 50–59 (APC = −5.8, *p* = 0.032) and those aged 85+ (APC = −2.7, *p* = 0.033) (Table 3). However, all age-specific APCs were negative, decreasing in absolute value with increasing age through the 80–84 age group. Over the same period, the ASIR for stage IV prostate cancer for men aged 50–74 increased at an annualized rate of 3.7% (*p* = 0.010); among men aged 75 and older, the increase was 3.1% per year (*p* < 0.001) (Table 3, Figure 5A,B). Increases in stage IV incidence rates were also observed for each 10-year age group, ranging from an annualized increase of 2.6% among men aged 50–59 (*p* = 0.022) to 4.1% among men aged 60–69 (*p* < 0.001). The incidence rate of unknown stage cases decreased in every age group from 2010 to 2017 (all *p* < 0.001). Though not directly comparable with the data presented for 2010 to 2017, stage IV rates for 2018 to 2021 were higher except in 2020 (Figure 6A,B).

### 3.5. Net Survival Trends

**Figure 7:**
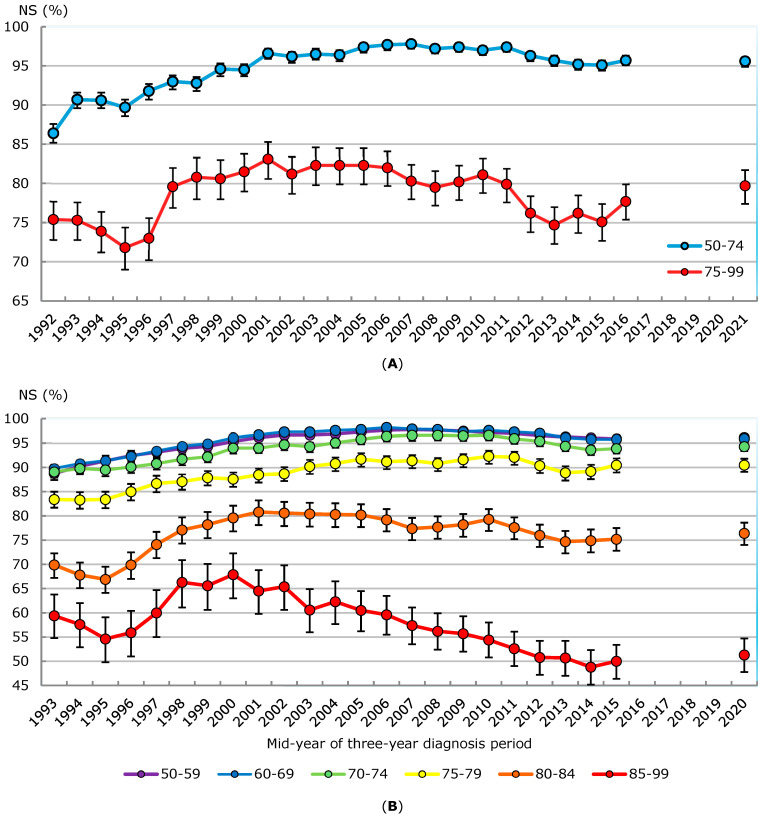
(**A**) Five-year prostate cancer net survival (NS) estimates by diagnosis year, males, ages 50–74 versus 75–99, Canada excluding Quebec, 1992 to 2021. (**B**) Five-year age-specific prostate cancer net survival (NS) estimates, males, Canada excluding Quebec, overlapping three-year periods from 1992–1994 to 2019–2021. NS for 2021 and 2019–2021 was predicted using period analysis. Quebec was excluded because prostate cancer case ascertainment in this province significantly differed from other jurisdictions for much of the study period, and because cases diagnosed in this province from 2018 onward had not been submitted to the Canadian Cancer Registry at the time the file used to perform the analysis was created. Cases diagnosed in Nova Scotia in 2020 and 2021 were also similarly unavailable. Here, 95% confidence intervals are denoted by vertical bars overlaid on the trend lines. Sources: Statistics Canada, Canadian Cancer Registry death-linked file (1992–2021), life tables.

Prostate cancer five-year net survival among men aged 50–74 generally increased from the early 1990s to the mid-2000s, increasing from a low of 86.4% (95% CI: 85.2–87.6%) in 1992 to a peak of 97.8% (97.2–98.3%) in 2007 (Figure 7A, Supplementary Table S1). After a plateau of several years, net survival declined from 97.4% (96.8–97.9%) in 2011 to 95.1% (94.4–95.7%) in 2015. The predicted five-year net survival estimate for cases diagnosed in 2021 was 95.6% (94.9–96.1%). Among men diagnosed with prostate cancer aged from 75 to 99, five-year net survival increased from 71.8% (69.0–74.4%) in 1995 to a peak of 83.1% (80.6–85.3%) in 2001. Net survival remained above 80% through the 2000s but then declined from 81.1% (78.8–83.2%) in 2010 to 75.1% (72.7–77.4%) in 2015. The predicted net survival estimate for 2021 of 79.7% (77.4–81.7%) was higher than the estimate for 2015 (*p* = 0.005).

An analysis of differences in survival between 2010 and 2011 and between 2016 and 2017 demonstrated that apparent decreases in unadjusted net survival over this period could be attributed to changes in the stage-mix composition of cases (Supplementary Table S2). Among prostate cancer cases identified as stage I-IV or unknown, five-year net survival decreased by 1.5 percentage points among men aged 50–74 (*p* < 0.001), and 3.0 percentage points among men aged 75–99 (*p* = 0.010), during this period. However, when the estimates for 2010-to-2011 were adjusted to the stage mix of the 2016-to-2017 cases, these survival decreases were transformed into non-statistically significant differences of 0.3 (*p* = 0.352) and −0.3 (*p* = 0.758) percentage points, respectively.

Three-year overlapping five-year net survival estimates among men in their 50s and 60s closely mirrored each other throughout the study period (Figure 7B, Supplementary Table S1). Following 8–9 percentage point increases since 1992 to 1994, net survival peaked at 97.8% (97.3–98.2%) among men aged 50–59 in 2006-to-2008, and at 98.2% (97.7–98.6%) among men aged 60–69 in 2005-to-2007. A period of decline followed; net survival had decreased to just under 96% in both age groups by 2014-to-2016. A similar picture was observed among men aged 70–74 with typically slightly lower five-year survival, most apparent in the late 1990s and early 2000s. Net survival peaked in this age group at 96.6% in several different three-year periods, first in 2006-to-2008 and lastly in 2009-to-2011. However, by 2014-to-2016 it had declined to 93.9% (92.9–94.8%).

Compared to men aged 70–74, five-year prostate cancer net survival among men aged 75–79 was 3.5–6.5 percentage points lower for each three-year period studied. Net survival peaked at 92.3% (90.9–93.5%) in 2009-to-2011, following a nine-percentage point increase from the early 1990s. By 2012-to-2014, net survival had declined to 88.9% (87.3–90.3%). For men diagnosed with prostate cancer at even older ages, five-year net survival increased more rapidly and peaked earlier. Following increases of 13–14 percentage points, net survival peaked in 2000-to-2002 at 80.8% (78.1–83.2%) among men aged 80–84, and in 1999-to-2001 at 67.9% (63.0–72.3%) among men aged 85–99. Subsequent declines in net survival were observed in both age groups, to 74.7% (72.3–76.9%) in 2012-to-2014 among men aged 80–84, and to 48.8% (45.2–52.3%) in 2013-to-2015 among men aged 85 to 99. The decline among men aged 80–84 was more pronounced after 2009-to-2011 than before.

**Figure 8:**
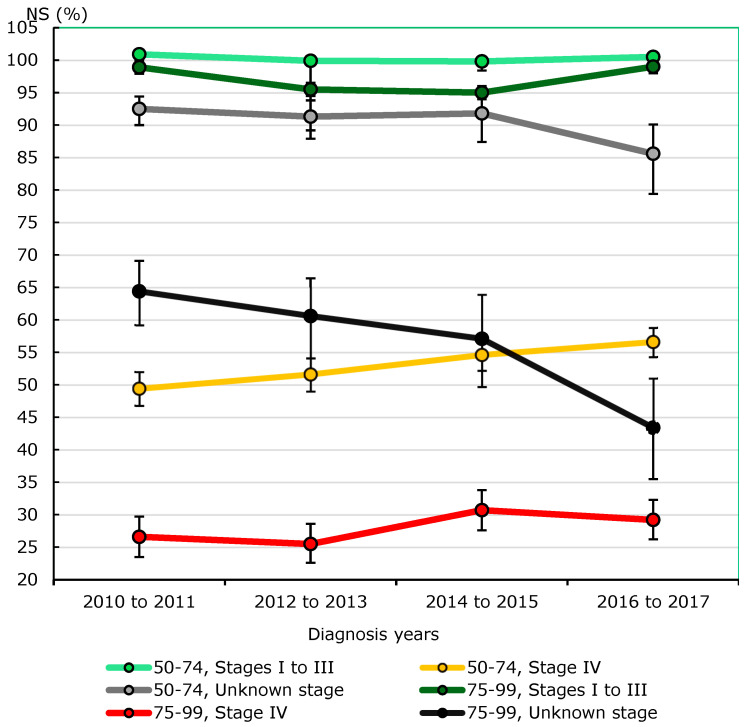
Five-year stage-specific prostate cancer net survival (NS), males, by age group, Canada excluding Quebec, 2010–2011 to 2016–2017. Notes: Quebec was excluded because cases submitted to the Canadian Cancer Registry for this period did not include information on stage at diagnosis. Prostate cancer cases were staged using the Collaborative Stage (CS) data collection system, and American Joint Committee on Cancer (AJCC) 7th Edition stage groups were derived using CS data/variables. Here, 95% confidence intervals are denoted by vertical bars overlaid on the trend lines. Sources: Statistics Canada, Canadian Cancer Registry death-linked file (1992–2021), life tables.

Among men aged 50–74 diagnosed with stage IV prostate cancer, five-year net survival increased from 49.4% (46.8–52.0%) in 2011-to-2012 to 56.6% (54.3–58.8%) in 2016-to-2017 (Figure 8). In comparison, stage IV net survival was lower among men aged 75–79, and a 3.2 percentage point increase over this period was not statistically significant (*p* = 0.233). For both age groups, net survival remained stable for stage I/II/III prostate cancer, while it was observed to decrease for cases with an unknown stage (50–74: *p* = 0.018).

Except where otherwise noted, all changes in net survival described above were highly statistically significant (*p* < 0.001).

## 4. Discussion

Prostate cancer incidence, stage, mortality, and net survival in Canada have changed relative to key shifts in prostate cancer screening recommendations in the US. Prostate cancer incidence rose sharply among men aged 50–84 before PSA screening was widely available. Incidence peaked at this time among men aged 70 and older, while the peak among men aged 50–69 was in 2007, after screening was accessible. Following USPSTF recommendations against screening in 2008 and 2011, the ASIR among men aged 50–74 dropped, and then subsequently leveled off starting in 2014. Mortality decreased after screening was widely available, but after guideline changes against screening, mortality stabilized among men aged 55–69 and decreased at a lesser pace among older men. For most age groups, much of the mortality reduction occurred before screening recommendations changed, despite many treatment advances occurring after this time. Concurrently with the period of flattened incidence and reduced mortality decline, the rate of stage IV cases increased significantly in all 10-year age groups. The five-year net survival peaked around the time of recommendations against screening and then declined because of a shift towards a greater proportion of stage IV (metastatic) cases.

Prostate cancer incidence trends in Canada (excluding Quebec) mirrored those observed in the US, with a similar increase after the introduction of screening, and a decrease associated with changes in USPSTF recommendations [36,65]. While incidence has been increasing in the US since 2014 [66], this has not been noted thus far in Canada. Both Canadian and US prostate cancer incidence rates dipped in 2020, likely because of interruptions in screening and reduced access to health care during the COVID-19 pandemic [67,68]. The rates of aggressive Gleason disease and stage IV cancer increased in the US after 2012, with metastatic prostate cancer increasing by 36.9% [37,43,65,69,70]. US prostate cancer mortality rates peaked in the early nineties, and then dropped by about half until after 2013, when mortality stagnated or even increased [21,38,71]. Mortality increases were more pronounced in black men and men over age 60 [38].

Similar increases in prostate cancer incidence prior to the introduction of screening were also observed in countries without screening, suggesting that other factors may be implicated in this rise [72]. The increasing use of PSA, initially for diagnosis and then gradually for screening, transrectal ultrasound-guided biopsies, and transurethral resections of the prostate may have all contributed [73]. Incidence in men aged 75 and older decreased after the introduction of screening, concurrent with a rise in incidence noted in men aged 50–74, suggesting that screening was not contributing to increased prostate cancer diagnosis in older men.

The pattern of prostate cancer incidence by age seen in 1984 could reflect a baseline for symptomatic presentation of clinically relevant cases, given there was no screening at this time. The natural history of prostate cancer is such that early-stage prostate cancers may develop into metastatic disease over a 10- to 15-year period [74]. The large burden of cases in older men in 1984 aligns with this natural history, suggesting increasing presentation of clinically significant prostate cancer with advancing age. The changing curve of incidence rate by age after 1984 likely reflects the impacts of screening on diagnosis, with a shift to diagnosis at an earlier age and a significant drop in cases in older men.

Prostate cancer mortality among men aged 50–74 dropped by over half after the advent of screening. Some of this mortality benefit was driven by therapeutic advances, such as the use of adjuvant luteinizing hormone-releasing hormone agonists in combination with radiation therapy. However, the adoption of multiple new therapies for patients with metastatic disease, such as chemotherapy, androgen pathway inhibitors, and radioligand therapy, occurred after recommendations against screening, resulting in improved survival for stage IV disease; but overall, a lesser mortality reduction than was seen after the introduction of screening. These findings are consistent with literature showing 85.4% of prostate cancer mortality reduction was due to increased diagnosis of early-stage disease with screening, and modeling where screening was found to decrease prostate cancer mortality by 45–70% from 1988 to 2016 [24,75]. These relative reductions have a large absolute effect, given the high number of deaths from prostate cancer—there were 512.6 deaths for every 100,000 men aged 75 and older at peak mortality. The high financial and personal cost of treatment for metastatic cases could potentially be reduced with screening, enabling diagnosis of prostate cancer at an earlier stage.

Slight declines in prostate cancer net survival estimates in Canada (excluding Quebec) after recommendations against screening may be attributed to changes in the stage distribution of cases—all stage-specific net survival estimates either remained steady or improved. In the setting of significant treatment advances, five-year net survival among stage IV cases in men aged 50–74 increased from 49.4% to 56.6% over the short period from 2010–2011 to 2016–2017. Net survival also increased, though statistically non-significantly, from 26.6% to 29.2% among older men, while it remained close to 100% among stage I-III cases for both age groups. However, the combined proportion of stage I–III cases declined from 2010 to 2017, resulting in fewer good prognosis cases, including presumably cases of overdiagnosis, which may have previously inflated survival. Over the same period, incidence rates of stage IV prostate cancer increased by 23% among men aged 50–74, by 32% among men aged 60–69, and by 21% among men aged 75 and older. Rates of unknown stage cases also dropped from 2010 to 2017, but over this period, the associated net survival decreased, suggesting that stage IV cases were constituting a larger component of these cases as time went on.

Changes in the stage distribution of prostate cancer cases after recommendations against screening, particularly the increased rate of stage IV disease, likely had an impact on the decreased prostate cancer mortality reduction, counteracting the benefits of treatment advances. The available stage data from 2018 to 2021 suggests that stage IV prostate cancer incidence rates may have continued to increase in more recent years. The presented rates for 2018 to 2021 may underestimate actual rates for the jurisdictions providing data, considering that only 77.9% of prostate cancer cases diagnosed among men aged 50 and older in this period had sufficient information to be categorized as stages I to IV. Additionally, the definition of stage IV disease in AJCC 8 is slightly more conservative than that in AJCC 7. The full impact of mortality related to increasing stage IV disease may not yet be realized as the median overall survival of metastatic prostate cancer with contemporary treatments is 57 months [76].

Concerns regarding the utility of PSA testing are primarily related to overdiagnosis and subsequent overtreatment. These are important concepts, as treatment side effects, such as incontinence and erectile dysfunction, can have profound impacts on quality of life. Overdiagnosis rates vary widely depending on age, Gleason score, and PSA, in one model ranging from 2.9 to 88.1% [77]. Although the drop in stage I cases suggests some overdiagnosis was likely ongoing, the different timing of peak incidence in men older and younger than 70 in this study suggests the “harvest” effect did not occur in older men, where overdiagnosis is more relevant. The continuous decline in incidence in men older than 70 after screening suggests that the ongoing diagnosis of prostate cancer in younger men may have found clinically relevant cases that did not then present symptomatically in those older than 70. The recent increase in incidence in men in their early eighties suggests this trend may be reversing, given a known PSA screening-related lead time of 5.5 to 6.3 years for white men and 7.7 years for black men [24,78]. It is important to note that although survival can be impacted by lead time bias, mortality is not. The joinpoint interval with the greatest mortality reduction (2002–2007, APC = −8.3) was observed among men aged 55–59. This suggests a strong benefit of screening and treatment advances in this group of younger men with potentially biologically aggressive disease for whom overdiagnosis is less relevant. Finally, mortality rates consistently declined after the onset of screening. However, despite multiple treatment advances, the pace of age-specific declines either diminished or leveled out after screening was no longer recommended. At the same time, the rate of diagnosis of stage IV prostate cancer increased. Together, this suggests that earlier detection with PSA was finding cases before they were metastatic, where treatment was potentially curative.

Overdiagnosis and overtreatment can be minimized by more targeted and nuanced use of PSA testing, and more careful selection of individuals for biopsy and treatment [6]. The Canadian Urological Association advises that testing intervals should be contingent upon PSA values, with screening only performed every four years in the setting of a lower risk PSA < 1 ng/mL, and more frequently in the context of a higher risk PSA of >3 ng/mL [27]. Longer screening intervals for individuals with low PSA values can reduce false positives by 50% and overdiagnosis by 27% [79]. PSA testing is only advised in individuals with a life expectancy greater than 10 years [27]. Contemporary diagnostic assessment for an abnormal PSA focuses on systematic harm reduction from overdiagnosis and overtreatment. Repeat PSA testing, the use of PSA density, and free to total PSA can eliminate unnecessary biopsies [33,80]. Risk assessment with MRI can successfully avoid biopsies in at least 28% of men with elevated PSA [81,82]. Use of targeted biopsy increases the detection of clinically significant cancer while decreasing the diagnosis of low-grade indolent cancer. Low-grade and some intermediate-grade prostate cancers can be managed with active surveillance, reserving definitive therapies until there are signs of increased disease activity or aggressivity. Ideally, practices to reduce harms from PSA screening would be incorporated in screening guidelines to maintain the benefit of early detection while reducing unnecessary biopsies and treatments.

PSA screening is variably funded across Canadian provinces and territories. In the absence of organized screening programs, Canadian men need to self-advocate, have a primary care provider order their PSA, and, in provinces with no access to screening, pay for their test. Significant correlations have been noted between men who accessed PSA screening and higher income, higher levels of education, non-smoking status, and attachment to a primary care provider [34]. There are also disparities in prostate cancer outcomes according to race and ethnicity, as black men are more likely to be diagnosed at younger ages with more aggressive disease and experience greater mortality than white men [83]. Before the advent of PSA screening, 20% of white and 40% of black men in the US had metastatic disease at diagnosis [24]. Indigenous men are also diagnosed with more aggressive disease and have increased rates of metastatic disease than their non-indigenous counterparts [84]. Minority populations, especially black men, have been shown to derive more benefit than white men from screening [24].

As this was an ecological study, observed associations reflect population level patterns. Trends may be influenced by unmeasured individual-level factors such as changes in MRI use, biopsy practices, adoption of active surveillance, evolving therapies, health care access, or interruptions related to the COVID-19 pandemic. The incidence data used in this study from 1992 onward originated from the CCR, one of the highest quality national population-based cancer registries in the world [85]. However, it was necessary to exclude incidence data from Quebec and from Nova Scotia from 2020 onward. Over the study period, the population of Quebec constituted about one-quarter of that of Canada. While we have no evidence to suggest that the prostate cancer incidence trends in Quebec differed from that of the rest of Canada, we cannot dismiss the possibility and the impact that potentially could have on the incidence trends reported herein. Stage-specific analyses commenced with the 2010 data year, as prostate cancer stage information was limited and not consistently available for most jurisdictions on the CCR in prior years. Stage systems changed from AJCC 7 to 8 in 2018 and along with data coverage differences precluded the direct comparison of stage-specific prostate cancer incidence rates between the 2010–2017 and 2018–2021 periods. PSA and Gleason values for prostate cancer cases were missing in a substantial proportion of cases, precluding their use. The CCR does not distinguish between cases detected through screening versus those that were symptomatically detected, and as such, we can only make inferences around relationships with screening. The availability of such information would facilitate a more direct study of prostate cancer screening and outcomes.

## 5. Conclusions

Prostate cancer screening has never been recommended in Canada by the CTFPHC, yet substantial opportunistic screening activity is ongoing, with over half of Canadian men over age 50 having accessed screening at some point in their lives. Canadian trends in prostate cancer incidence, mortality, stage distribution, and survival were associated with USPSTF PSA screening recommendations. The majority of prostate cancer mortality reduction occurred while screening was recommended, and rising stage IV rates after recommendations against screening can partially account for decreasing net survival—unadjusted for stage distribution—and flattening of mortality declines. While significant advances have been made in the treatment of metastatic disease, the rising incidence of stage IV cancers comes at a high personal cost and represents a substantial financial burden for our health care system. Screening enables earlier stage diagnosis with its inherent improved outcomes and lower costs and should be viewed in the context of ongoing innovations such as active surveillance and the use of prostate MRI to inform biopsy, which are reducing overdiagnosis and overtreatment. Currently, there is a burden on individuals to self-advocate, which can undermine health equity, particularly considering documented disparities in prostate cancer outcomes according to race. Organized and thoughtful screening may represent an opportunity to offer clear guidance on the appropriate ages and intervals for screening and diagnostic assessment for an abnormal PSA.

## Figures and Tables

**Table 1:** Trends in age-standardized prostate cancer incidence and mortality rates, males, Canada, 1984–2023.

	Incidence (Excluding Quebec)			Mortality		
Age Group	Trend Period	Annual Percent Change (95% Confidence Limits)	*p*-Value	Trend Period	Annual Percent Change (95% Confidence Limits)	*p*-Value
50+	1984–1993	5.8 (4.1, 9.1)	<0.001	1984–1994	1.3 (0.7, 2.0)	<0.001
	1993–2009	−0.2 (−0.8, 0.5)	0.554	1994–2012	−2.8 (−3.2, −2.6)	<0.001
	2009–2014	−7.1 (−8.5, −4.4)	0.001	2012–2023	−1.4 (−1.8, −0.6)	0.010
	2014–2022	0.9 (−0.5, 3.3)	0.154			
50–74	1984–1993	9.0 (6.9, 13.8)	0.001	1984–1988	3.8 (1.0, 5.7)	0.001
	1993–2007	1.8 (0.7, 2.9)	0.006	1988–1994	−0.8 (−4.4, 0.1)	0.077
	2007–2014	−5.9 (−8.8, −4.0)	0.005	1994–2010	−4.3 (−5.1, −1.3)	0.022
	2014–2022	0.2 (−1.6, 3.2)	0.687	2010–2023	−1.0 (−1.6, −0.3)	0.023
75+	1984–1992	3.2 (1.6, 5.6)	<0.001	1984–1995	1.4 (0.9, 2.0)	<0.001
	1992–2015	−3.2 (−3.7, −3.0)	<0.001	1995–2012	−2.5 (−3.1, −2.3)	0.001
	2015–2022	1.3 (−0.4, 3.6)	0.115	2012–2023	−1.6 (−1.9, −0.7)	0.006

Notes: Quebec was excluded from incidence because prostate cancer case ascertainment in this province significantly differed from other jurisdictions for much of the study period, and because cases diagnosed in this province from 2018 onward had not been submitted to the Canadian Cancer Registry at the time the file used to perform the analysis was created. Cases diagnosed in Nova Scotia in 2020 and onward were similarly unavailable. The annual percent changes were calculated using the Joinpoint Regression Program, where incidence data for 2020 were removed as an anomaly. Rates are age-standardized to the 2021 Canadian Standard Population. Sources: Canadian Cancer Registry (1992–2022), National Cancer Incidence Reporting System (1984–1991) at Statistics Canada, and Canadian Vital Statistics Death database at Statistics Canada.

**Table 2:** Trends in age-specific prostate cancer incidence and mortality rates, males, Canada, 1984–2023.

	Incidence (Excluding Quebec)			Mortality		
Age Group	Trend Period	Annual Percent Change (95% Confidence Limits)	*p*-Value	Trend Period	Annual Percent Change (95% Confidence Limits)	*p*-Value
50–54	1984–2001	11.3 (9.9, 16.5)	<0.001	1984–2023	−1.6 (−2.3, −0.8)	0.001
	2001–2007	3.4 (−2.4, 8.8)	0.179			
	2007–2022	−4.2 (−5.8, −3.3)	0.001			
55–59	1984–2001	8.9 (7.8, 10.8)	0.001	1984–2002	−2.0 (−2.9, 1.0)	0.086
	2001–2009	1.0 (−1.1, 4.1)	0.285	2002–2007	−8.3 (−11.0, −3.2)	0.028
	2009–2014	−8.0 (−9.8, −5.0)	0.007	2007–2023	0.1 (−1.2, 3.1)	0.669
	2014–2022	−0.9 (−2.5, 2.1)	0.555			
60–64	1984–1993	11.0 (8.5, 16.6)	0.001	1984–1988	5.4 (0.0, 9.1)	0.051
	1993–2007	2.9 (1.7, 4.1)	0.004	1988–2011	−3.6 (−4.7, −3.3)	<0.001
	2007–2015	−6.1 (−9.2, −4.5)	0.006	2011–2023	−0.5 (−1.6, 1.5)	0.488
	2015–2022	0.1 (−2.1, 3.0)	0.818			
65–69	1984–1993	9.2 (7.1, 13.8)	<0.001	1984–1993	2.2 (0.7, 4.4)	0.004
	1993–2007	1.6 (0.5, 2.7)	0.008	1993–2011	−4.4 (−5.7, −3.9)	<0.001
	2007–2014	−6.0 (−8.9, −4.1)	0.001	2011–2023	−0.6 (−1.8, 1.3)	0.359
	2014–2022	0.7 (−0.9, 3.6)	0.290			
70–74	1984–1993	7.4 (5.6, 10.8)	0.002	1984–1988	4.9 (1.4, 7.9)	0.010
	1993–2009	−1.0 (−1.7, 0.0)	0.055	1988–2000	−2.4 (−3.2, −1.6)	<0.001
	2009–2014	−6.5 (−8.3, −3.3)	0.002	2000–2006	−6.7 (−8.4, −4.7)	0.009
	2014–2022	1.6 (−0.2, 4.9)	0.062	2006–2023	−1.6 (−2.0, −0.8)	0.008
75–79	1984–1992	5.9 (3.4, 9.8)	<0.001	1984–1995	0.5 (−0.4, 1.6)	0.274
	1992–2016	−2.7 (−3.5, −2.4)	<0.001	1995–2013	−4.1 (−5.0, −3.7)	<0.001
	2016–2022	3.2 (−0.3, 6.0)	0.066	2013–2023	−1.3 (−2.4, 1.0)	0.126
80–84	1984–1992	3.9 (2.6, 5.9)	<0.001	1984–1992	2.1 (0.8, 3.9)	0.001
	1992–1997	−5.8 (−6.8, −3.5)	0.002	1992–2023	−2.6 (−2.8, −2.5	<0.001
	1997–2016	−3.0 (−3.5, −2.1)	<0.001			
	2016–2022	2.0 (0.1, 4.0)	0.041			
85+	1984–1992	0.5 (−0.9, 2.9)	0.442	1984–1995	1.7 (0.9, 2.9)	<0.001
	1992–2015	−3.7 (−4.3, −3.5)	<0.001	1995–2023	−1.5 (−1.7, −1.4)	<0.001
	2015–2022	−0.8 (−2.4, 1.1)	0.370			

Notes: Quebec was excluded from incidence because prostate cancer case ascertainment in this province significantly differed from other jurisdictions for much of the study period, and because cases diagnosed in this province from 2018 onward had not been submitted to the Canadian Cancer Registry at the time the file used to perform the analysis was created. Cases diagnosed in Nova Scotia in 2020 and onward were similarly unavailable. The annual percent changes were calculated using the Joinpoint Regression Program, where incidence data for 2020 was removed as an anomaly. Sources: Canadian Cancer Registry (1992–2022), National Cancer Incidence Reporting System (1984–1991) at Statistics Canada, and Canadian Vital Statistics Death database at Statistics Canada.

**Table 3:** Trends in stage-specific prostate cancer incidence rates, males, selected age groups, Canada excluding Quebec, 2010–2017.

	Stage I to III		Stage IV	
Age Group	Annual Percent Change (95% Confidence Limits)	*p*-Value	Annual Percent Change (95% Confidence Limits)	*p*-Value
50–74	−4.9 (−11.2, 1.7)	0.137	3.7 (1.0, 6.9)	0.010
75+	−2.4 (−6.7, 2.0)	0.252	3.1 (1.6, 5.0)	<0.001
50–59	−5.8 (−11.1, −0.6)	0.032	2.6 (0.3, 5.2)	0.022
60–69	−4.8 (−10.2, 0.9)	0.093	4.1 (3.1, 5.3)	<0.001
70–79	−3.3 (−8.8, 2.5)	0.239	3.9 (1.6, 6.7)	<0.001
70–74	−3.7 (−10.4, 3.5)	0.275	4.5 (−0.6, 10.9)	0.079
75–79	−2.8 (−8.0, 2.6)	0.267	3.8 (1.2, 6.7)	0.006
80+	−2.3 (−5.4, 1.0)	0.174	3.0 (1.5, 4.6)	<0.001
80–84	−1.7 (−4.8, 1.7)	0.279	3.4 (2.3, 4.6)	<0.001
85+	−2.7 (−5.1, −0.2)	0.033	2.2 (−0.2, 5.0)	0.068

Notes: Quebec was excluded because, at the time of analysis, cases submitted to the Canadian Cancer Registry by this province did not include staging information. Trend analyses for the 50–74 and 75+ age groups were conducted using rates age-standardized to the 2021 Canadian Standard Population. Source: Canadian Cancer Registry (1992–2022) at Statistics Canada.

## Data Availability

Data is available through the Research Data Centres program at Statistics Canada https://www.statcan.gc.ca/en/microdata/data-centres. Accessed on 1 November 2025.

## Supplementary Materials

The following supporting information can be downloaded at: https://www.mdpi.com/article/10.3390/curroncol32120669/s1, Figure S1. (a) Prostate cancer age-standardized and age-specific incidence rates, males, Canada excluding Quebec, 1984–2022. (b) Prostate cancer age-standardized and age-specific mortality rates, males, Canada, 1984–2023. Supplementary Table S1. Five-year age-specific prostate cancer net survival and 95% confidence interval (CI), males, by diagnosis period, Canada. Supplementary Table S2. Differences in five-year prostate cancer net survival (NS), unadjusted versus adjusted for stage mix, males, Canada excluding Quebec, 2010–2011 and 2016–2017 periods.
